# Sex Differences in Response to Viral Vector Vaccines—Implications for Future Vaccine Design

**DOI:** 10.1111/imr.70098

**Published:** 2026-01-30

**Authors:** Ilka Grewe, Tamara Zoran, Marylyn Martina Addo

**Affiliations:** ^1^ Institute for Infection Research and Vaccine Development (IIRVD) University Medical Center Hamburg Eppendorf (UKE) Hamburg Germany; ^2^ First Department of Medicine, Division of Infectious Diseases University Medical Center Hamburg‐Eppendorf Hamburg Germany; ^3^ Bernhard‐Nocht‐Institute for Tropical Medicine, Department for Clinical Immunology of Infectious Diseases Hamburg Germany; ^4^ German Center for Infection Research (DZIF), Partner Site Hamburg‐Lübeck‐Borstel‐Riems Hamburg Germany

**Keywords:** MVA, MVA‐MERS‐S, nonreplicating vaccines, replicating, sexual dimorphism, viral vector vaccines

## Abstract

Vaccination represents one of the most impactful public health achievements, preventing 3.5 to 5 million deaths annually according to estimates of the World Health Organization. Yet, recent outbreaks of emerging and reemerging infectious diseases highlight the need for rapid and strategic vaccine development using vaccine platforms technologies. Sexual dimorphism in vaccine‐induced immune responses has received significant attention in recent years. To ensure vaccine safety and efficacy across sexes, sex‐based differences should be considered in vaccine design, dosing, and regimen. Evidence on many traditional vaccines, such as the inactivated influenza vaccine, shows a female bias in innate and adaptive immune responses following vaccination. Thus, it has long been suggested that females universally develop stronger humoral and cellular immune responses to vaccines compared to males. However, compared to traditional vaccines, studies investigating sex differences following vaccination with next‐generation platforms, such as viral vector vaccines, remain limited. This review provides an overview of clinical observations of sex differences in responses to replication‐competent and replication‐deficient, recombinant and nonrecombinant viral vaccines. Additionally, we describe the current state of knowledge on mechanisms of sex‐based differences in immune responses and possible implications for future vaccine design.

## 1 Introduction

Vaccination represents one of the most impactful public health achievements, substantially reducing morbidity and mortality of infectious diseases worldwide [1]. The World Health Organization (WHO) estimates that vaccines prevent 3.5 to 5 million deaths annually, and an estimated 154 million deaths were prevented through the essential program of immunization (EPI) since 1974 [2, 3]. The eradication of smallpox and the near‐eradication of polio are key milestones that have been achieved through vaccination, demonstrating the profound global health impact of vaccines. However, the 21st century has seen the emergence and reemergence of infectious diseases, including Ebola virus, severe acute respiratory syndrome coronavirus (SARS‐CoV), Middle East respiratory syndrome coronavirus (MERS‐CoV), severe acute respiratory syndrome coronavirus 2 (SARS‐CoV‐2) and Mpox outbreaks, which continue to pose a significant threat to global health. These events have highlighted the need not only for effective vaccines, but for vaccine platforms that can be rapidly adapted, manufactured, and deployed during public health emergencies. Historically, most licensed vaccines consisted of either a whole attenuated version of a pathogen, inactivated pathogens or pathogen fragments (reviewed in [4]). During the last two decades, the development of next‐generation vaccines, based on platform technologies has been increasingly applied in order to combat epidemic or pandemic outbreaks (reviewed in [5]). Incorporating an antigen from an emerging pathogen into a carrier system, such as a viral vector or mRNA lipid nanoparticle, enables the rapid development of new vaccine candidates based solely on the antigen's sequence information. This means that there is no need for production of high amounts of the emerging pathogen under the respective biosafety level, such as biosafety level 3 for SARS‐CoV‐2 in the beginning of the COVID‐19 pandemic or biosafety level 4 for Ebola virus. Instead, vaccine platform technologies allow rapid and high‐scale manufacturing of vaccines against new antigens, often with established manufacturing processes. Another advantage of platform technologies is their already known safety profile. The safety profile of each vaccine is unique and needs to be evaluated individually for regulatory approval. However, growing experience with vaccine platforms could potentially enable faster evaluation and approval in future, particularly in the event of a public health emergency (reviewed in [6, 7]). Consequently, next‐generation vaccines have gained importance in vaccine development and strategies for epidemic preparedness.

Until now, vaccine development mostly follows a “one‐size‐fits‐all” strategy, even though it is well recognized that individual factors such as age, underlying health conditions, concurrent infections, the microbiome, sex, and gender can significantly influence vaccine responses. In certain specific circumstances such as in the context of hemodialysis or advanced age, adapted higher doses of vaccines have been implemented, for example, high‐dose influenza vaccines for individuals ≥ 60 [8, 9].

Sex and gender have been identified as fundamental variables influencing vaccine outcomes. The term sex refers to biological characteristics such as chromosomes, hormones, and anatomy while gender refers to identity, behaviors, and roles that societies associate with being female, male, or gender diverse. Both biological and sociocultural factors influence susceptibility to and severity of a range of infections, as well as vaccine immunogenicity, efficacy, and reactogenicity, sometimes rendering it difficult to differentiate between primarily sex‐specific and rather gender‐specific effects (reviewed in [10, 11, 12]). Most studies reviewed in this manuscript did not specify whether cis‐ or transgender women and men were included, and many may not clearly distinguish between sex and gender. When reporting biological differences between men and women, these studies presumably refer to cisgender participants. Despite the SAGER guidelines, recent reports indicate that the clinical trial and research communities still do not use the terms sex and gender consistently or appropriately [13].

This review focuses primarily on biological differences between individuals assigned female or male at birth, while also acknowledging that gender‐related behavioral and sociocultural factors may influence these outcomes and warrant consideration.

Increasing evidence from both clinical and preclinical studies demonstrates that females and males differ in the magnitude and quality of innate and adaptive immune responses to vaccination, often resulting in differences in vaccine immunogenicity and adverse events (reviewed in [12, 14, 15, 16, 17]). For a long time, it has been considered that females universally mount stronger innate, humoral, and cellular immune responses to vaccines compared to males. This has been shown for many vaccines, including those against influenza, yellow fever, hepatitis A and B, as well as the live‐attenuated vaccine against measles, mumps, and rubella. For influenza vaccines, particularly pronounced sex differences in vaccine‐induced immune responses have been demonstrated. Across numerous studies, females consistently exhibit stronger humoral and cellular immune responses, but also higher numbers of adverse events following immunization with inactivated influenza vaccines [18, 19, 20, 21]. A prospective, randomized trial assessing different doses of the trivalent inactivated influenza vaccine revealed that women mounted significantly higher antibody responses than men, with geometric mean titers in women receiving a half‐dose approximating or exceeding those of men given the full dose [18]. The authors suggested that a lower trivalent inactivated influenza vaccine dose could be sufficient for females and future vaccine strategies could include dose adjustment for sex. Also following other inactivated and protein vaccines, such as the hepatitis A virus (HAV) vaccine, consisting of formalin inactivated virus, and the hepatitis B virus (HBV) vaccine consisting of oligomers of the HBV surface antigen, females consistently mount higher antibody responses [22, 23, 24, 25, 26, 27, 28, 29]. In conclusion, sex differences in immune responses to inactivated virus vaccines, protein subunit vaccines as well as to several live attenuated vaccines have been studied for many years and higher immune responses in females seemed relatively consistent across different vaccines (reviewed in [14]).

Given the rather recent emergence of vaccine platforms such as viral vector and mRNA vaccines, to date less is known about sex differences in the immune responses they elicit. During the SARS‐CoV‐2 pandemic, the widespread use of viral vector and mRNA vaccines provided a unique opportunity to advance understanding of sex bias in vaccine responses. Although biological sex is a variable collected in all clinical trials and studies, data were rarely disaggregated or analyzed by sex, by both vaccine producers and clinical studies [30, 31]. Therefore, despite the availability of large datasets that could have advanced our understanding of sex bias in the context of newer vaccine platforms, the effect of sex was mostly not systematically studied, further complicating a comprehensive comparison of available vaccine platforms. In a systematic review of COVID‐19 clinical trials at the beginning of the pandemic, Heidari and colleagues reported that 24% of the trials reported sex disaggregated data and only 13% discussed the implications of observed sex bias [30]. In a recent review on mRNA COVID‐19 vaccines, only 9% of publications reporting vaccine effectiveness for licensed or approved COVID‐19 vaccines provided sex‐disaggregated data, while 28.3% did not even report the sex distribution of their study populations. Assessing sex‐specific responses to COVID‐19 vaccination was further hindered by substantial heterogeneity in study designs, target populations, vaccine types, and vaccination status [31].

Despite the challenges discussed above, numerous studies have explored sex differences in vaccine efficacy, adverse events, and immunogenicity in the context of COVID‐19 vaccines (reviewed in [32, 33]). A comprehensive comparison of COVID‐19 vaccines is beyond the scope of this review. The efficacy of mRNA vaccines (BioNTech/Pfizer BNT162b2 or Moderna mRNA‐1273), ChAdOx1 nCoV‐19 (AstraZeneca), and Ad26.COV2.S (Janssen), as well as inactivated SARS‐CoV‐2 vaccines, has been proven in both females and males [34, 35]. Sex‐related disparities have been mostly noted in adverse events and antibody responses [35, 36]. Briefly, mRNA vaccines have been associated with an elevated risk of myocarditis, predominantly in young males following BNT162b2 or mRNA‐1273 vaccination, although the absolute risk remains far lower than that linked to SARS‐CoV‐2 infection [37]. Viral vector vaccines against SARS‐CoV‐2, in particular ChAdOx1 nCoV‐19 (Oxford/AstraZeneca), have been associated with rare vaccine‐induced thrombotic thrombocytopenia (VITT), with early reports suggesting higher occurrence in young women, while later analyses did not confirm a clear sex predominance [38, 39, 40, 41, 42]. Following the initial dose of mRNA vaccination (BNT162b2 or mRNA‐1273), elderly females developed higher neutralizing antibody titers than elderly males, but this sex difference disappeared following the second dose [43]. Complementing these findings, Oyebanji et al. (2025) observed that elderly women exhibited stronger antibody responses than elderly men after COVID‐19 vaccination, yet these sex disparities in antibody titers diminished following booster administration [44]. In response to ChAdOx1 nCoV‐19 vaccination, females developed higher anti‐spike IgG titers compared to males, but no sex differences were observed in neutralizing antibodies [45]. In a recent systematic review of mRNA COVID‐19 vaccines, Bachmann et al. (2025) reported that evidence for sex bias in vaccine‐induced immune responses is not consistent across studies. While many investigations found that females generally mounted stronger antibody responses following mRNA SARS‐CoV‐2 vaccination compared to males, several studies reported no detectable sex differences [36].

Taken together, SARS‐CoV‐2 vaccines developed using new‐generation platform technologies illustrate the ongoing challenges of integrating biological sex as a research variable and suggest that sex biases in vaccine‐induced immune responses may vary across vaccine platforms. These observations indicate that sex bias in immune responses may be more complex and more nuanced than previously appreciated and warrant greater attention in future research. Moreover, the availability of vaccines targeting the same antigen but delivered through different carrier systems may provide a unique opportunity to advance our understanding of how sexual dimorphism shapes vaccine‐induced immune responses.

Unlike traditional vaccine platforms, viral vector vaccines may introduce distinct biological factors that could influence the mechanisms underlying sex‐related differences in vaccine‐induced immune responses. For example, replication competency of a given viral vector may alter antigen load and duration of exposure, potentially influencing sex‐differential innate immune activation, reactogenicity, and the quality of adaptive immune responses. Similarly, preexisting immunity to viral vector backbones may differ by sex due to biological or exposure‐related factors, with implications for vaccine performance. Given the increasing global relevance of viral vector vaccines for pandemic preparedness and emerging infectious disease control, understanding sex‐specific responses to these platforms is not only scientifically important but also essential for ensuring equitable and optimized vaccine strategies. Therefore, in this review, we provide an overview of current evidence on sex differences in immune responses to nonrecombinant live viral vaccines and recombinant viral vector vaccines. Subsequently, we explore mechanisms of sex‐based differences in immune responses to these vaccines.

## 2 Live Attenuated and Viral Vector Vaccines

Whole viral pathogens were the first vaccines applied, starting with the inoculation of dried and pulverized material from smallpox lesions (also termed variolation) leading to protection against smallpox [46]. Attenuation of whole pathogens led to vaccines with more acceptable safety profiles, while inducing long‐lasting immune responses [47]. Today, many live attenuated vaccines, such as vaccines against yellow fever, measles, mumps, rubella and varicella as well as the live attenuated influenza vaccine are widely used [48]. In addition, several live attenuated viral vaccines are deployed as viral vector backbones for new recombinant vaccine candidates. A first recombinant viral vector vaccine candidate was developed as early as 1984, by inserting a Hepatitis B antigen into a nonpathogenic vaccinia virus strain, and showed efficacy in chimpanzees challenged with HBV [49, 50]. However, it took almost three more decades until the first viral vector vaccines successfully underwent clinical trials in humans [51, 52, 53, 54]. Especially, the efficacy of the Vesicular Stomatitis Vector vaccine expressing the Ebola virus glycoprotein VSV‐EBOV during the 2013–2016 epidemic represented a breakthrough in the development of viral vector vaccines with VSV‐EBOV (Ervebo) representing the first ever licensed viral vector vaccine in 2019 [55, 56]. Subsequently, in 2020, the COVID‐19 pandemic led to a rapid increase in the development of viral vector vaccines (reviewed in [5]).

The ability of viral vectors to infect host cells, which then express the target antigen and present it through the major histocompatibility complex (MHC), enables strong humoral and cellular immune responses [57]. Viral vectors can be either replication‐competent or replication‐deficient. Replication‐competent viral vectors bear similar advantages as live‐attenuated vaccines, as the longer‐lasting presence of the antigen induces stronger and potentially longer‐lasting immunity. While replication within host cells can enhance antigen availability and immunogenicity, this benefit must be weighed against the potential for increased adverse effects or disease caused by uncontrolled replication, particularly in immunocompromised individuals. Nonreplicating viral vector vaccines generally have more favorable safety profiles while still administering the antigen directly into the host cells. Furthermore, viral vectors induce innate immune responses without the need for adjuvant [58, 59] (reviewed in [60]). The impact of viral vector backbones, their replication competency, and innate immune responses towards them on sex‐specific immune outcomes remain incompletely understood to date (Table 1).

**TABLE 1:** Reviewed studies on viral vector vaccines, reporting stratified data on vaccine efficacy, immunogenicity, and/or adverse events for sex.

Vector	Target pathogen	Dependent measures stratified for sex	Sex bias	References
VSV	EBOV	Binding antibodies Neutralizing antibodies	F = M/F > M (heterogenous data)	[61, 62, 63, 64]
YF‐17D	YF	Neutralizing antibodies	F > M	[65]
BERNA‐YF	YF	Neutralizing antibodies	F < M	[65]
RKI‐YF	YF	Neutralizing antibodies	F < M	[65]
ChAd	SARS‐CoV‐2	Efficacy (determined by occurrence of SARS‐CoV‐2 symptomatic illness and positive RT‐PCR) Binding antibodies	F = M/F > M (heterogenous data)	[45, 66]
ChAd	EBOV	Seroconversion rate	F = M	[63]
MeV	MeV	Binding antibodies Neutralizing antibodies	F = M/F > M (heterogenous data)	[67, 68, 69]
Ad5	HIV	Antigen‐specific T‐cell response (ELISpot)	F > M	[70]
		Enhanced HIV‐acquisition^a^	F < M	[71, 72, 73]
Ad5	EBOV	Adverse event: Elevated body temperature/fever (37.6°C–39.0°C)^b^	F < M	[74]
Ad5	SARS‐CoV‐2	Vaccine efficacy (determined by occurrence of SARS‐CoV‐2 symptomatic illness and positive RT‐PCR)	F < M	[75]
Ad26	SARS‐CoV‐2	Efficacy (determined by occurrence of SARS‐CoV‐2 symptomatic illness and positive RT‐PCR)	F = M	[76]
Ad26	ZIKV	Neutralizing antibodies	F > M	[77]
MVA	Smallpox/MPXV	Binding antibodies Neutralizing antibodies	F < M	[78]
MVA	MERS‐CoV	Neutralizing antibodies	F = M	Figure 1
Dryvax	Smallpox	Neutralizing antibodies	F > M	[80]

^a^ Following vaccination with the Ad5 gag/pol/nef HIV‐1 vaccine, enhanced HIV‐1 acquisition was observed. This effect was greater for men than for women and was strongest in Ad5‐seropositive uncircumcised male vaccinees.

^b^ Following vaccination with the Ad5‐EBOV, males compared with females had a higher risk for grade 2 fever, defined in the study as body temperature between 37.6°C and 39.0°C, measured by thermometers with an infrared sensor.

## 3 Replicating Nonrecombinant and Recombinant Viral Vaccines

### 3.1 VSV‐Based Vaccines

The development and clinical evaluation of VSV‐EBOV during the 2013–2016 epidemic represents a significant milestone in viral vector vaccine technology. This live‐attenuated, replication‐competent vaccine expresses the EBOV glycoprotein and showed efficacy in nonhuman primates already in 2005 [81, 82]. VSV‐EBOV was first applied in humans as an experimental postexposure prophylaxis following accidental exposure due to a needlestick injury during an animal experiment in the biosafety level 4 laboratory in Hamburg [83]. During the 2013–2016 epidemic, VSV‐EBOV has demonstrated safety, immunogenicity, and efficacy in clinical studies, including in ring vaccination trials [55, 56, 62, 63, 84, 85, 86, 87]. Further studies during outbreaks in the DRC confirmed its effectiveness, leading to regulatory approval by the European Medicines Agency (EMA) and the U.S. Food and Drug Administration (FDA) in 2019 [88, 89]. Data on sex differences in immune responses to VSV‐EBOV are scarce and most clinical trials leading to licensure of the vaccine did not stratify immunogenicity outcomes by sex.

Our group contributed to the early clinical evaluation of VSV‐EBOV as part of the WHO‐led VEBCON consortium (VSV‐EBola CONsortium). Four phase I trials were conducted in parallel in Lambarene, Gabon (PACTR2014000089322), Kilifi, Kenya (NCT02296983), Geneva, Switzerland (NCT02287480), and at our site in Hamburg, Germany (NCT02283099) [56, 90]. In the Hamburg open‐label study, we assessed safety and immunogenicity of ascending doses, including analysis of T cell responses, innate immune responses, miRNA, and VSV‐specific cellular and humoral responses [91, 92, 93, 94]. Due to the small cohort within each dose group and biological heterogeneity, we were not able to comprehensively study sex differences. Similarly, within each of the other VEBCON studies alone, sex differences were not systematically analyzed. However, in a 5‐year follow‐up to investigate the durability of antibody responses, sex was not found to predict the magnitude of responses as measured by ELISA, and no sex differences in the persistence of antibody responses were observed [61].

A subsequent phase 2 placebo‐controlled trial conducted in Liberia analyzed safety and immunogenicity of the VSV‐EBOV vaccine and an Ebola vaccine based on the ChAd vector. In this study, immunogenicity was similar in all analyzed subgroups that were defined according to age, sex, and previous contact with a person who had Ebola virus disease [63]. Another phase 2 trial included front‐line workers and evaluated humoral and cellular immune responses. They found no differences between females and males in mean concentrations of IgG against EBOV GP [62]. However, a post hoc analysis of three phase 2 clinical trials, including both phase 2 trials mentioned above, investigated the influences of sex and age on immunogenicity of the VSV‐EBOV Ebola vaccine [64]. While age did not have a significant impact on immunogenicity in the pooled analysis, biological sex did show an effect. Total IgG antibody levels (EU/mL), measured by Glycoprotein‐ELISA, and neutralizing antibody responses assessed by the plaque reduction neutralization test (PRNT), were higher in females than in males as early as day 28 and remained elevated through day 365. However, it should be noted that none of the studies included in this post hoc analysis was prospectively designed to evaluate sex differences in immune responses. A retrospective case–control study conducted during the 2018–2020 Ebola virus disease outbreak in the Democratic Republic of Congo did not find differences between sexes in vaccine effectiveness. By comparing vaccination rates between Ebola virus disease‐positive cases and matched Ebola virus disease‐negative controls, the study estimated the real‐world effectiveness and found comparable VSV‐EBOV‐induced protection against Ebola virus disease in females and males [95].

In conclusion, the evidence on sex differences in VSV‐EBOV induced humoral and cellular immune responses and consecutive protection against Ebola virus disease is heterogeneous with some data pointing towards a female bias and some studies finding comparable immunogenicity outcomes between the sexes.

Building on the successful development of VSV‐EBOV, several other recombinant vaccines have been developed on the VSV vector backbone (reviewed in [96]). VSV‐based vaccine candidates against filoviruses encoding the Marburg virus Glycoprotein, the Sudan virus glycoprotein and Bundibugyo Ebola virus glycoprotein showed efficacy against lethal challenge with respective viruses in animal models [97, 98, 99, 100]. Moreover, a VSV‐based Lassa virus (LASV) vaccine, which expresses the LASV preglycoprotein complex induces strong cellular and humoral immune responses in nonhuman primates despite genetic variability of LASV isolates [101]. Additionally, VSV‐based vaccine candidates against Nipah virus, Crimean‐Congo Hemorrhagic Fever virus, Zika virus and MERS‐CoV have been designed and are in various stages of development [102, 103, 104, 105, 106]. Furthermore, a VSV‐SARS‐CoV‐2 vaccine was evaluated in a phase 1/2 safety and immunogenicity trial [107]. In light of new VSV‐based vaccine candidates potentially entering clinical trials soon, understanding immune responses towards them and potential sex differences in the efficacy of VSV‐based vaccines is imperative.

### 3.2 Yellow Fever Virus‐Based Vaccines

The yellow fever virus strain 17D (YF‐17D) is a live‐attenuated virus vaccine that induces temporal viremia and protective antibody responses lasting for decades after a single vaccination [108, 109].

The YF‐17D strain in use today was initially developed from the wild‐type yellow fever virus Asibi‐strain through repeated passage on mouse embryo tissue and chicken embryo tissue in 1937 [110, 111]. Ever since, YF‐17D has been widely used for immunization against yellow fever, resulting in enormous experience with this vaccine. While YF‐17D represents an enormous success in terms of induction of strong immune responses and long‐lasting protection against yellow fever, rare severe and potentially lethal complications have led to recommendations of YF‐17D being restricted to young and immunocompetent individuals [112, 113, 114, 115, 116]. Acute viscerotropic disease following yellow fever vaccination (YEL‐AVD) is a severe complication characterized by rapid onset of systemic illness resembling wild‐type yellow fever, often progressing to multiorgan failure and is associated with a mortality rate of approximately 50% [117]. YF vaccine‐associated neurotropic disease (YEL‐AND) typically presents with neurological symptoms such as encephalitis, meningoencephalitis, or Guillain–Barré–like syndromes, usually occurring within days to weeks after vaccination [118]. Both severe complications are associated with age > 60 years and a compromised immune system, but can also occur in immunocompetent individuals below the age of 60 years [117, 118].

Notably, the incidence of both YEL‐AVD and YEL‐AND is significantly higher in men compared to women with a male to female ratio between 2.75: 1 and 6: 1 [118, 119, 120]. This is in line with the wild‐type yellow fever virus also causing more severe disease in males compared to females [121]. However, even more interestingly, sex imbalance in the incidence of YEL‐AVD changes over the life course. Lindsey et al. found in an analysis of all YEL‐AVD cases reported to the U.S. Vaccine Adverse Event Reporting System (VAERS) between the years 2002 and 2007, that 75% of cases under age 40 were female, compared to 11% of cases ≥ 40 years [119]. This finding was confirmed by an analysis of a different dataset from the Centers for Disease Control and Prevention [122]. On the other hand, females have reported more local reactions such as injection site erythema, pruritus and pain [119]. Since VAERS relies on passive reporting, differences in reporting behaviors between females and males should also be taken into account [119].

Underlying mechanisms of sex differences in response to the yellow fever vaccine, including more local reactions in female vaccinees and higher incidence of YEL‐AVD in young females and older males, are not yet understood. Data on differences in humoral immune responses to YF‐17D between males and females are inconsistent [14, 65]. A study by Pfister et al. [65] found that females develop higher antibody titers than males in response to the commercially available YF‐17D strain (Stamaril). However, the same study observed that males develop significantly higher antibody titers in response to the sub strains BERNA‐YF (Flavimun) and the Robert Koch Institute sub strain RKI YF [65]. A comparative study of YF‐17D vaccines (ARILVAX and YF‐VAX) found that male gender was associated with higher antibody responses [123]. Evidence on differences in innate immune responses following YF‐17D point towards a female bias. In a reanalysis of publicly accessible microarray data of healthy volunteers vaccinated against yellow fever virus, Klein et al. found a stronger transcriptional response following vaccination in females than in males [14]. In this analysis, 660 genes were differentially expressed at either day 3, 7, or 10 post vaccination in females compared to only 67 differentially expressed genes in males. In particular, female vaccinees had greater transcriptional activity of genes involved in toll‐like receptor signaling and the IFN pathway [14]. Given that inflammatory cytokines and chemokines are believed to mediate adverse effects of YF‐17D, the stronger innate immune response seen in females may underlie their higher incidence of local reactions early post vaccination [14].

Understanding the mechanisms and sex differences in response to YF‐17D gains new importance, since the viral vaccine is also investigated as a recombinant viral vector platform for several other antigens [124, 125, 126, 127, 128]. The first recombinant vaccine developed on a YF‐17D backbone encodes the structural proteins prM and E of Japanese encephalitis virus [125]. Based on these first experiences of deploying YF‐17D as a viral vector backbone for recombinant vaccines, the dengue virus vaccine candidate Dengvaxia has been developed. Following clinical evaluation of this tetravalent vaccine candidate in 26 clinical trials including more than 41,000 volunteers, Dengvaxia has been approved in several countries for individuals who have previously had a dengue infection [53, 54, 126]. Additionally, viral vector vaccines on a YF‐17D backbone against West Nile virus (WNV) and ZIKV have been developed [128, 129, 130, 131]. To date, there is no reported evidence on sex differences in adverse events or immunogenicity induced by the recombinant YF‐17D vaccines against Japanese encephalitis virus, dengue virus, WNV or ZIKV.

### 3.3 Chimpanzee Adenovirus (ChAd)‐Based Vaccines

Chimpanzee adenovirus (ChAd) vectors represent a widely used recombinant viral backbone for vaccine development and have been applied for vaccines against a wide range of pathogens, including Ebola virus, MERS‐CoV, Plasmodium falciparum, and most prominently SARS‐CoV‐2 [63, 132, 133, 134, 135, 136, 137, 138, 139]. ChAd vectors are nonenveloped viruses. Therefore, antigens such as membrane glycoproteins are not displayed on the virion surface but are instead produced at high levels following entry and transduction of host target cells [132].

A substantial body of evidence on immune responses to ChAd‐based vaccines has emerged during the COVID‐19 pandemic. ChAdOx1 nCoV‐19 (Oxford/AstraZeneca) encodes the nonstabilized wildtype version of the SARS‐CoV‐2 spike protein.

Although clinical trials leading to regulatory approval of ChAdOx1 nCoV‐19 included females and males, data were in most studies initially not stratified for sex [133, 134, 135]. Results from a large phase 3 clinical study showed that the efficacy of the ChAdOx1 nCoV‐19 vaccine was similar in male and female vaccinees [66]. An analysis of the effects of age and sex on immune outcomes of 15,169 volunteers enrolled in two randomized controlled trials of ChAdOx1 nCoV‐19 found greater anti‐spike IgG titers in females than in males [45]. However, no sex differences were observed in pseudoneutralization titers, antibody isotypes, or antibody subclasses [45].

During vaccination rollout in the COVID‐19 pandemic, sex differences in adverse events following ChAdOx1 nCoV‐19 vaccination led to concerns about vaccination of population groups of a specific age and sex. The Vaccine‐induced thrombotic thrombocytopenia (VITT) is a rare but severe vaccination‐induced autoimmune thrombosis syndrome. Clinical presentation includes thrombosis, often in unusual sites, and many patients present with more than one site of thrombosis [38, 39, 40]. In early reports of VITT, cases appeared to occur more frequently in women and younger adults [40, 41, 42]. Later analyses did not confirm a strong female predominance [38, 40, 41, 42]. The initial observation of more young females being affected may have reflected the vaccination demographics at that time, particularly the high proportion of young female healthcare workers vaccinated during the initial rollout [38].

In addition to the recombinant SARS‐CoV‐2 vaccine, several other recombinant ChAd vector vaccines have been developed and evaluated in clinical trials [63, 132, 136, 137, 138]. A recombinant ChAd3 vector vaccine expressing the EBOV glycoprotein has been evaluated as a single dose and as a heterologous booster following vaccination with the MVA‐based Ebola vaccine [63, 137, 138]. A study comparing the ChAd3‐EBOV vaccine to the VSV‐EBOV vaccine found no sex differences in immune responses following either vaccine [63]. Furthermore, ChAd vector vaccine candidates against MERS and Plasmodium falciparum have been evaluated [136, 139]. Reported data have mostly not been stratified for sex. Besides, to date it remains unclear if other ChAd‐based viral vector vaccines induce similar complications as VITT observed following ChAdOx1 nCoV‐19 vaccination.

### 3.4 Measles Viral Vector Vaccines

First licensed in 1963, the live‐attenuated measles virus (MeV) has emerged as a promising viral vector platform for recombinant vaccine development [140]. Its long history of safe use, combined with its ability to induce strong humoral and cellular immune responses, makes it an interesting delivery system for heterologous antigens. Although preclinical evaluation is complicated by the fact that MeV naturally only infects primates, limiting the range of suitable animal models, several MeV‐based vaccine candidates have nonetheless progressed successfully through the early stages of preclinical and clinical development [140]. Notably, the MeV‐based Chikungunya vaccine has completed Phase II clinical trials, demonstrating safety and immunogenicity, and is now advancing to Phase III evaluation [141]. This highlights the potential of the MeV platform for delivering safe and effective vaccines in humans.

Sex‐related differences in immune responses to the standard nonrecombinant measles vaccine have been documented for several decades [68]. Studies have indicated that in childhood and adolescence, females typically generate higher antibody levels than males [15]. Furthermore, patterns of long‐term measles antibody persistence vary by sex and age. While adult males (40–49 years) generate higher IgG antibodies against measles during mid‐adulthood, females show comparatively higher antibody levels in older age (≥ 60 years) [69]. Of note, the live attenuated measles vaccine is today mostly applied as the combined measles, mumps, and rubella vaccine, rendering it difficult if sex differences in adverse events and innate immune responses are attributable to MeV or to the other live attenuated components of the vaccine [8, 142]. The influence of sex on immune responses to recombinant vaccines based on the measles virus remains largely unexplored. Given the established patterns observed with the traditional measles vaccine, similar sex differences in immunity between males and females are conceivable.

## 4 Nonreplicating Recombinant and Nonrecombinant Viral Vaccines

### 4.1 Human Adenovirus‐Based Vaccines

Human adenovirus (Ad), especially the serotypes 5, 26, and 35, are used as recombinant viral vector backbones for several vaccine antigens [71, 75, 76, 143, 144, 145, 146, 147, 148]. Adenoviruses are a diverse family of double‐stranded DNA viruses that can cause respiratory, ocular, and gastrointestinal infections and were first discovered in 1953 in tonsil and other adenoid tissue of children [149, 150]. The adenoviral genome is divided into early and late regions, depending on the time point of gene expression relative to the onset of viral DNA replication [151, 152]. Early genes encode transcription units, while late genes encode Ad structural proteins [153]. In order to insert a transgene into the viral vector, one or more early AdV genes are deleted, rendering most adenoviral vectors replication‐incompetent [153, 154, 155]. Depending on the exact early region that is being deleted, there are a few adenoviral vectors that are replication competent [154, 155, 156, 157, 158].

Early human adenoviral vector vaccines were developed using human adenovirus serotype 5 (Ad5), a virus with a high seroprevalence in humans [132]. However, preexisting antibodies against Ad5 are common and might reduce humoral and cellular responses generated by the vaccine—a challenge that is overcome when using human adenovirus serotype 26 or 35 (Ad26 and Ad35, respectively) [159].

Several phase 1 and 2 studies investigated replication‐incompetent Ad5 as a viral vector vaccine candidate for HIV‐1 [71, 143, 144].

A trivalent HIV‐1 clade B vaccine, which contains Ad5 vectors expressing clade B gag, pol, and nef epitopes, was investigated in a phase 2b study in South Africa (Phambili trial) [143]. While the vaccine did not prevent HIV‐1 infection, immune responses recognized HIV‐1 clade B and C subtypes, thereby demonstrating cross‐clade reactivity. A subsequent analysis of predictors of immune responses found that female sex was associated with higher immune responses to the clade C‐pol antigen [70]. The Phambili trial suspended enrollment and vaccination after the interim analysis of another phase 2 trial of the Ad5 gag/pol/nef HIV‐1 vaccine (the Step trial) found a potential enhanced HIV‐1 acquisition in Ad5‐seropositive uncircumcised male vaccinees compared to unvaccinated controls [71]. Long‐term follow‐up of both the Phambili and the Step trial confirmed the Ad5 gag/pol/nef HIV‐1 vaccine induced enhancement of HIV‐1 acquisition among male vaccinees [72, 73]. Interestingly, the follow‐up of the Phambili trial also found an increased HIV‐1 acquisition risk in female vaccine recipients, but this effect was weaker for women than for men [72]. Consequently, Ad5 was not further developed as a viral vector vaccine for HIV and these findings have profoundly affected the development of an HIV‐vaccine.

However, recombinant replication‐incompetent Ad5 was further investigated as a viral vector vaccine against EBOV and SARS‐CoV‐2 during respective public health emergencies of international concern [75, 145, 146, 147, 148]. In a clinical trial of a recombinant Ad5 vaccine expressing the EBOV glycoprotein, males had a higher risk of developing fever post vaccination compared to females [74, 145]. Ad5‐SARS‐CoV‐2 in combination with Ad26‐SARS‐CoV‐2 is part of a heterologous vaccine regimen against SARS‐CoV‐2 (licensed under the brand name Sputnik V, produced by the Gamaleya Research Institute of Epidemiology and Microbiology in Russia) [148]. A phase 3 study investigating a single‐dose Ad5 vectored vaccine expressing the SARS‐CoV‐2 spike protein and including 21,250 participants in the primary efficacy cohort, found that vaccine efficacy was higher in men (65.8%) than women (40.0%) [75]. However, a smaller phase 2 study did not find any sex differences in RBD‐specific antibodies, neutralizing antibodies or T cell responses following a single shot of the same vaccine [160]. Taken together evidence points towards a probable male bias in immune responses to Adenovirus 5 vaccines, a finding which was not observed for Ad26‐based viral vectors.

The Ad26‐SARS‐CoV‐2 vaccine (also Ad26.COV2.S; produced by Janssen, a subsidiary of Johnson & Johnson) is a recombinant nonreplicating adenovirus 26–vectored vaccine encoding the SARS‐CoV‐2 spike protein [76]. A single dose provides 76.7% efficacy against severe–critical COVID‐19 disease [76]. For Ad26‐SARS‐CoV‐2, no differences between immune responses in male and female vaccinees were identified in a phase 3 clinical trial [76]. Following vaccination with Ad26‐SARS‐CoV‐2, cases of VITT have been reported (reviewed in [38]). However, the majority of VITT cases occurred following ChAdOx1 nCoV‐19 vaccination, and only 12% of cases occurred in temporal context to Ad26‐SARS‐CoV‐2 (reviewed in [38]). A replication‐incompetent Ad26 viral vector vaccine encoding the ZIKV membrane and envelope proteins induced higher antibody titers in women than in men [77].

### 4.2 MVA‐Based Vaccines

Modified Vaccinia virus Ankara (MVA) serves as a promising recombinant vector against multiple pathogens and has recently gained new importance in its nonrecombinant form as a vaccine against Mpox virus (MPXV) [161]. As the bivalent smallpox and Mpox vaccine MVA is produced by Bavarian Nordic as MVA‐BN; licensed under the brand names Imvanex [Europe], Imvamune [Canada], and JYNNEOS [United States]. MVA was derived from the Ankara strain of the vaccinia virus through extensive serial passage in primary chicken embryo fibroblast cultures. This process has led to the loss of its ability to replicate productively in mammalian cells due to the inability of virion assembly, while retaining its ability to enter any cell and start its life cycle, resulting in unimpaired expression of viral early and intermediate genes [162]. The replication‐deficient strain maintained strong immunogenic properties, including the induction of humoral and cellular responses [163]. Furthermore, later studies demonstrated that MVA mediates a strong induction of type I Interferons and lacks several immune evasion factors [164, 165, 166, 167, 168, 169]. MVA was originally developed as a highly attenuated smallpox vaccine and early clinical evaluations in 1974 showed a favorable safety profile, leading to a first marketing authorization in 1977 and more than 120,000 people being vaccinated with the MVA smallpox vaccine until 1980 without any severe complications ([170, 171], reviewed in [162]). This is of importance since replication‐competent smallpox vaccines based on the vaccinia virus, which were critical for the eradication of smallpox, are associated with a less favorable safety profile. A first‐generation replication‐competent live vaccinia virus vaccine was produced based on the New York City Board of Health (NYCBOH) strain of vaccinia virus and was licensed under the brand name Dryvax. Later, this vaccine was replaced by the second‐generation smallpox vaccine ACAM2000, which also uses a live vaccinia virus but is grown in Vero cells. Potentially fatal adverse events include postvaccinal encephalitis, myopericarditis, and generalized vaccinia through uncontrolled replication and blood‐borne dissemination of vaccinia virus [172, 173, 174]. Smallpox was successfully eradicated in 1980 as declared by the World Health Assembly, with the last natural case occurring in Somalia in 1977 [175, 176, 177]. Consequently, smallpox vaccination of the general population was discontinued, but both replication‐competent smallpox vaccines and highly attenuated vaccines, such as MVA, are kept in stockpiles for use in immunocompetent and immunocompromised individuals, respectively, in case of a new outbreak or in preparation for potential bioterrorism threats [178, 179].

While for most viral vaccines, stronger humoral immune responses have been observed in females compared to males, this might not be the case for MVA vaccines. To our knowledge, the first evidence of sex differences following vaccination with the MVA‐based smallpox vaccine (Imvamune) was reported by Troy et al. (2015) [78]. Their meta‐analysis of three randomized clinical trials demonstrated that Imvamune does not elicit female‐biased humoral responses; instead, males developed antibody titers approximately 27% higher compared to females [78]. Although human studies on sex differences in immune responses to MVA‐based smallpox vaccines remain limited, animal data similarly suggest a male bias in humoral immunity [180]. Notably, these findings differ from sex differences observed in replication‐competent smallpox vaccines. A study by Kennedy et al. (2009) analyzed sex, age, and ethnic differences in immune responses following immunization with the live‐virus vaccinia‐derived vaccine Dryvax [80]. After adjusting for time since vaccination, neither age nor ethnicity significantly influenced immune responses, whereas sex did, with females exhibiting significantly higher vaccinia‐neutralizing antibody titers than males [80].

Due to the unprecedented global outbreak of MPXV clade 2b in 2022, the MVA vaccine and, consequently, also observed sex differences following MVA vaccination gained new importance. In response to the outbreak, nonrecombinant MVA was authorized for vaccination against MPXV clade 2b by the European Medicines Agency under “exceptional circumstances” [181]. The 2022 outbreak of MPXV clade 2b disproportionally affected communities of men having sex with men (MSM), leading to stigmatization of the LGBTQ+ community [182, 183, 184, 185, 186, 187, 188]. In addition to contact with skin lesions as in previous outbreaks, mucosal transmission through sexual contact, particularly associated with high viral loads in the anal region, oral cavity, and semen, has been identified as a principal mode of transmission for the 2022 outbreak of clade 2b [186, 187]. Consequently, populations at risk, who were vaccinated against MPXV with nonrecombinant MVA, mostly included men [161]. Thus, actual data on vaccine efficacy against MPXV in females were scarce, and studies did not stratify immune responses for sex [161].

This became particularly relevant when a subsequent Mpox Public Health Emergency of International Concern was declared by the WHO on Aug 14, 2024, due to an outbreak of clade 1b MPXV in the Democratic Republic of the Congo and neighboring countries [189, 190, 191]. Current evidence suggests that, for the first time, sexual transmission also played a role in the clade 1b outbreak, since 13% of infected individuals were female sex workers and 40% of all adults infected reported transactional sex [191]. However, household contacts as a potential source accounted for 50% of infections [191]. Unlike the outbreak of clade 2b, the clade 1b outbreak in the Democratic Republic of Congo affected women and men equally, as reported in a prospective cohort study by Brosius et al. (2025) [191]. Thus far, to our knowledge, no study has evaluated whether MVA induces comparable correlates of protection in females and males during the more recent MPXV clade 1b outbreak.

Apart from its significance as a nonrecombinant viral vaccine against smallpox and MPXV, MVA has been further developed as a promising recombinant viral vector against multiple pathogens (reviewed in [162]). The first MVA‐based recombinant vaccine was created in 1994, expressing genes encoding the hemagglutinin and nucleoprotein of the influenza A virus [192]. A recombinant MVA vaccine encoding the Ebola virus glycoprotein (MVA‐BN‐Filo) was the second viral‐vector vaccine to ever be approved and is currently recommended for use within a heterologous immunization regimen against Ebola [193, 194]. Furthermore, MVA has been evaluated in early clinical trials as a recombinant viral vector vaccine against HIV, RSV, CMV, and Plasmodium falciparum [195, 196, 197, 198, 199, 200, 201, 202, 203, 204].

#### 4.2.1 MVA‐MERS‐S: A Novel Vaccine Candidate Against MERS

**FIGURE 1:**
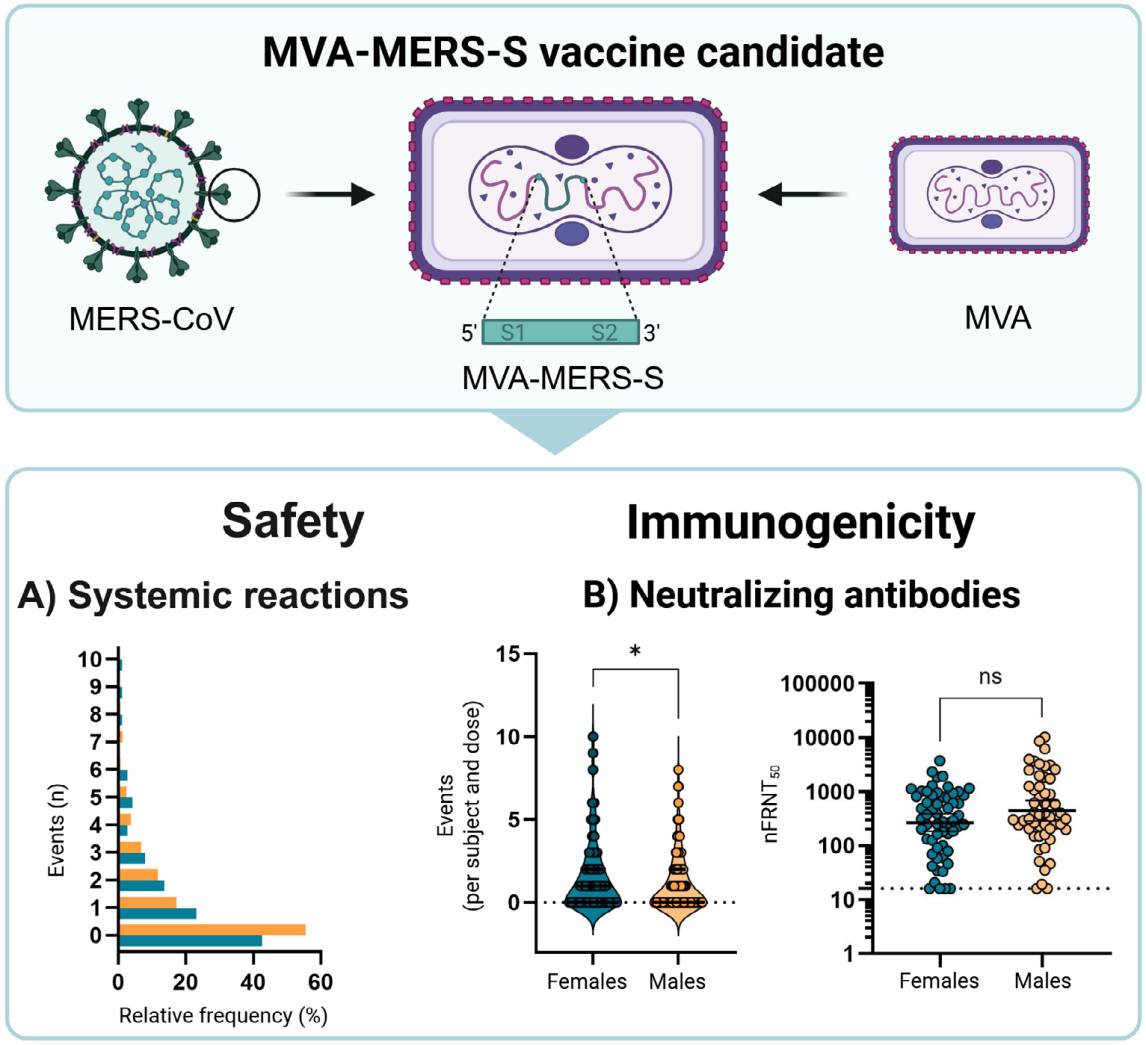
Summary of current evidence on the safety and immunogenicity of the MVA‐MERS‐S vaccine candidate against Middle East respiratory syndrome (MERS). Nonreplicating, MVA‐based vaccine encodes the MERS‐CoV spike protein. (A) Violin plots demonstrating systemic reactions per subject and dose, reported after administration of MVA‐MERS‐S and placebo. (B) Neutralizing antibodies, quantified 28 days after booster vaccination with MVA‐MERS‐S (pooled data from all investigated clinical arms for day 28 after booster vaccination). Data are shown as geometric mean titers with 95% confidence intervals. The Mann–Whitney test was used to investigate significance (ns, not significant; *, *p* < 0.05). Female individuals are shown in green and male individuals in orange. Figure created by the authors from original data partially reported by Raadsen et al. [79].

MVA‐MERS‐S is a recombinant MVA‐based vaccine candidate against the Middle East respiratory syndrome (MERS), expressing the full‐length spike (S) protein of MERS‐CoV (Figure 1), showing promising results in early clinical trials [79, 205]. The S protein is a highly immunogenic viral protein and the main target of most currently developed MERS‐CoV vaccine candidates [205]. To date, there is no licensed vaccine available against MERS, although several next‐generation vaccine platforms are currently in development and in clinical evaluation [79]. Furthermore, sex differences in immune responses to vaccines against MERS have not been systematically investigated, most likely because only smaller scale Phase 1 clinical studies have been performed to date. Safety and immunogenicity following MVA‐MERS‐S vaccination were recently evaluated in clinical trials, phase 1a and 1b [79, 205]. In brief, we reported favorable safety profiles with no serious adverse events reported following vaccination [79, 205]. We showed that MVA‐MERS‐S vaccination induces antibodies with both neutralizing and Fc‐mediated effector functions [79, 206]. The second dose of MVA‐MERS‐S elicited strong induction of neutralizing and MERS‐CoV‐S1 binding antibodies. The booster vaccination was safe, well‐tolerated, and significantly increased antibody titers [79, 205, 207]. Finally, we showed that the vaccine candidate also induces cellular immune responses by the subsequent production of antigen‐specific B and T cells [205, 208].

The double‐blinded, placebo‐controlled, randomized phase 1b clinical trial of the MVA‐MERS‐S vaccine candidate provided a better opportunity to examine sex differences than the earlier MVA‐MERS‐S phase 1a trial, due to the larger number of participants receiving the vaccine. We reported that vaccination with MVA‐MERS‐S did not lead to sex bias in local reactions [79]. Interestingly, females reported a higher number of systemic reactions after receiving either vaccine candidate or the placebo (Figure 1) [79]. Among reported systemic reactions were fatigue, myalgia, abdominal pain, arthralgia, nausea, and vomiting [79]. Overall, the investigation of humoral responses demonstrated that MVA‐MERS‐S vaccination does not lead to stronger antibody responses in females. Twenty‐eight days after the booster vaccination, GMT antibody levels were comparable across all treatment groups, irrespective of vaccine dose. Pooled analyses of S1 MVA‐MERS‐CoV‐specific binding antibodies showed significantly higher titers in males (data not shown). Similarly, the investigation also indicated a trend towards higher neutralizing antibody titers in males compared to females, although this difference did not reach statistical significance (Figure 1). These findings are consistent with the studies on the nonrecombinant MVA‐based smallpox vaccine Imvamune described above, which also demonstrated higher binding antibody responses in male than in female vaccine recipients [78, 180].

Although early‐phase clinical trials often enroll small cohorts, limiting the ability to analyze sex differences amid biological heterogeneity, available evidence on sex biases in these trials underscores the importance of investigating sex bias in response to vaccination, already in early stages of clinical trials.

## 5 Biological Mechanisms of Sex Bias in Vaccine‐Induced Immune Responses

As vaccine‐induced protection relies on a coordinated response including multiple components of the immune system, sex differences in various pathways, cellular and humoral responses ultimately lead to differences in reactogenicity and immunogenicity outcomes [14, 16, 17]. To better understand the role of biological sex in shaping sex‐specific immune responses, it is essential to not only stratify safety and immunogenicity data by sex but also to investigate the underlying mechanisms of sexual dimorphism.

Firstly, viral tropism, host cell entry, and viral sensing critically shape subsequent immune responses to viral vector vaccines [57, 132]. Live viral vaccines mostly induce stronger T cell responses compared to protein vaccines [57]. Hence, sex differences in T cell immunology play a crucial role for sex bias in viral vector vaccine efficacy [209]. Antibodies can neutralize pathogens directly or facilitate their elimination through mechanisms such as phagocytosis, complement activation, or recruitment of natural killer (NK) cells [210, 211]. These nonneutralizing functions depend on interactions between the antibody Fc region and Fc receptors on innate immune cells, triggering downstream immune responses [211]. Sex differences in both neutralizing and nonneutralizing antibodies have been described [18, 212, 213]. Additionally, innate immune responses to the viral vector may influence adaptive immune responses towards the target antigen [57]. Evidence across various diseases and vaccines found sex differences in innate immune responses [14, 17, 207, 214, 215]. Sex differences in the above‐described immune responses to vaccines are shaped by a complex interplay of several factors, including sex chromosomes, other genetic and epigenetic factors, sex hormones, and environmental factors such as microbiota [14, 15, 36, 216].

### 5.1 Genetic Factors

Differences in sex chromosomes between females (XX) and males (XY) play a critical role in sex bias in immune responses following vaccination. The X chromosome is larger than the Y chromosome and encodes more immune‐relevant proteins and microRNAs (miRNAs), which are important posttranscriptional regulators of immune responses [217]. Although in females one X chromosome undergoes dosage compensation via X‐chromosome inactivation (XCI), several immune‐relevant genes escape this silencing, leading to higher expression in XX individuals [218, 219].

Higher expression in female plasmacytoid dendritic cells (pDCs) due to escape from XCI has been reported for genes such as *TLR7*, *CYBB*, *RPS6KA3*, *BTK*, and *IL13RA1*. Moreover, pDCs with *TLR7* XCI escape had higher expression of IFNα/β, which are crucial in the immune response against viruses [219]. CXCR3 escape from XCI in T cells has been reported after infection with Leishmania, explaining the higher expression levels observed in females compared to males [218]. Since both DCs and T cells are important for vaccine‐induced responses, XCI might be one of the factors contributing to the female bias observed for some vaccines.

Emerging evidence suggests that miRNAs, small noncoding RNAs, may serve as valuable biomarkers for predicting both innate and adaptive immune responses. Several studies have demonstrated associations between miRNAs and various immunological outcomes, including humoral responses, cytokine production, and adverse events for different vaccines such as COVID‐19, influenza, and Ebola vaccines [220, 221, 222]. However, studies that report miRNA expression profiles by sex remain limited. In a recent study of healthcare workers vaccinated with the SARS‐CoV‐2 mRNA vaccine, Anticoli and colleagues [222] reported higher expression levels of miR‐221‐3p and miR‐148a‐3p in vaccinated females, while miR‐155‐5p was more highly expressed in vaccinated males. These findings highlight the need for further research to elucidate sex‐specific patterns of miRNA expression following vaccination and to explore the full potential of miRNAs as immune regulators, predictive biomarkers, and noninvasive biomarkers for the generation of effective vaccine‐induced responses.

### 5.2 Estrogens

Circulating sex hormone levels, as well as the presence and distribution of hormone receptors, which function as transcriptional regulators in immune cells, play a critical role in shaping immune responses [212]. The interplay between sex hormones and immune responses is complex. Few studies to date have investigated the association between vaccine‐induced responses and sex hormones; therefore, the exact role of sex hormones in responses to vaccines remains not well understood [223, 224]. Estrogens have been widely documented to enhance humoral responses, whereas male androgens are generally associated with immunosuppressive effects [223, 225, 226, 227].

Estradiol is the dominant biologically active estrogen during reproductive years and is primarily produced by the ovaries, whereas estrone, mainly derived from the adrenal glands, increases in concentrations during reproductive senescence. Estrogen signaling occurs through the nuclear receptors ERα and ERβ, as well as the membrane‐associated G‐protein–coupled estrogen receptor (GPER/GPR30). Both nuclear and membrane bound receptors are expressed on various innate and adaptive immune cells (reviewed in [228, 229]). Estradiol directly influences multiple immune cell types (reviewed in [228, 229]). In dendritic cells (DCs), exposure to estrogen increases their production of IL‐6, IL‐8, and monocyte chemoattractant protein‐1 (MCP‐1) [230]. Besides, estrogen enhances the ability of DCs to activate T lymphocytes [230]. Furthermore, estrogen receptor signaling promotes dendritic cell differentiation [231]. Of note, the influence of estradiol on dendritic cells (DCs) has been studied primarily in vitro and in vivo in mice, with a limited number of human studies showing similar findings [231, 232]. Modulation of effector functions of DCs by estrogens has been suggested as a factor contributing to the sex differences in disease severity observed in Covid‐19 (reviewed in [233]). However, to our knowledge, no studies investigated if estrogen receptor signaling in DCs plays a role in initial sex bias in immune responses following different Covid‐19 vaccination. Following rabies vaccination of estrogen‐deficient mice, activation and recruitment of DCs to the lymph nodes was significantly lower compared to mice with normal estrogen levels [226]. In monocytes and macrophages, estradiol promotes an anti‐inflammatory phenotype. The hormone inhibits inflammatory gene expression by controlling NF‐kappaB intracellular localization and decreases the production and secretion of IL‐6 following stimulation with LPS [234, 235, 236]. An in vitro study found that estradiol reduces inflammation of human macrophages induced by anti‐SARS‐CoV‐2 IgG, a mechanism associated with hyperinflammation in severe COVID‐19 patients [237]. To date, no studies have characterized the functional role of estrogen receptor signaling in monocytes and macrophages in the context of sex differences in immune responses towards vaccines. T cells express both nuclear estrogen receptors ERα and Erβ. Overall, CD4^+^ T cells express higher levels of ERα than ERβ, whereas CD8^+^ T cells exhibit comparable levels of both receptors (reviewed in [229]). Estrogen receptor signaling in T cells has been well studied in autoimmune diseases, with ERα signaling generally being proinflammatory and Erβ signaling being anti‐inflammatory (reviewed in [229]). Less is known about the role of ERα and Erβ receptor signaling in T cells in vaccine‐induced immune responses. In female rhesus macaques, ovariectomy impairs T‐cell immunity. Ovariectomized female rhesus macaques of reproductive age have a higher frequency of CD4 naïve T cells compared to controls with intact ovaries. Meanwhile, ovariectomized aged female rhesus macaques had increased frequency of differentiated CD4 memory T cells. Following MVA vaccination, ovariectomized animals of all ages presented delayed and reduced T‐cell proliferation as well as lower frequencies of vaccine‐specific cytokine‐producing T cells compared with ovary‐intact controls [238]. In B cells, estrogen receptor signaling regulates proliferation, differentiation, and functionality (reviewed in [229]). The above‐described studies investigating the effects of estrogen deficiency on rabies and MVA vaccination in mice and rhesus macaques, respectively, also found reduced B cell proliferation, activation, and recruitment following vaccination [226, 238].

The influence of estradiol on immunity has been researched intensively in influenza vaccination studies. Both mice and human data have demonstrated a positive correlation between estradiol levels and influenza‐specific antibody responses in females [223]. In mice, estradiol improved antibody responses following influenza vaccination [239, 240]. Evidence in mice further demonstrates that estrogen deficiency weakens cellular immune responses to rabies vaccination. While estrogen‐deficient mice immunized against rabies generate protective antibody responses, antibody titers decline more rapidly than in mice with normal estrogen levels [226]. Moreover, cellular and humoral immune responses following vaccination with MVA were reduced in female rhesus macaques with ovariectomy compared to controls with intact ovaries [238]. These divergent findings might reflect that the influence of estradiol on vaccine‐induced immunogenicity might differ for distinct vaccine types and antigens.

Sex hormone levels vary throughout life, contributing to different sex biases in immune responses, depending on age. Moreover, fluctuations in estradiol and progesterone across the menstrual cycle and throughout life influence immune responses in women [224, 241, 242]. Elderly individuals generally mount weaker antibody responses than younger adults, and sex bias in vaccine‐induced responses has been suggested to diminish with age [223, 243]. Adult females of reproductive age develop stronger antibody responses to influenza vaccination compared with elderly females and males [223]. In aged mice, supplementation with estradiol improved antibody responses following influenza vaccination, which were comparable to antibody responses in adult mice [240]. However, human studies on estradiol supplementation are more equivocal, as both enhanced and unaltered antibody responses to influenza vaccination in postmenopausal women have been reported [244, 245]. A retrospective analysis found that estradiol supplementation in postmenopausal women reduced the fatality rate of Covid‐19 compared to postmenopausal women who did not receive hormone therapy [246]. To our knowledge, no studies investigated the effect of estradiol supplementation on the immunogenicity of SARS‐CoV‐2 vaccines. Further research is required to determine whether estradiol supplementation in women with estradiol deficiency could enhance antibody responses to vaccines against different antigens and based on next‐generation vaccine platforms.

### 5.3 Testosterone and Other Androgens

In contrast to estradiol, testosterone has often been described to exert an immunosuppressive effect. Testosterone can be converted to estrogens or to other androgens, which signal through the androgen receptors (AR) (reviewed in [229]). T cell development is influenced by AR signaling in the thymus, by inhibition of positive selection, and AR signaling has been associated with suppression of CD4+ and CD8 + T cells [229]. A similar suppression role was observed for B cell functions, suggesting AR signaling has an important role in T and B cell activity in autoimmunity [229]. Human macrophages stimulated in vitro with testosterone showed decreased production of IFN‐γ and TNF [237]. Similarly, a decrease in proinflammatory cytokine secretion was observed for pDCs and neutrophils [229, 247]. Evidence indicates that testosterone enhances the expression of genes involved in lipid metabolism, and this induction in gene expression is linked to reduced antibody neutralization in men following influenza vaccination [227]. One example is gene *LTA4H*, encoding an enzyme that converts LTA4 to LTB4. Lipid mediator, LTB4, exhibiting both anti‐inflammatory and proinflammatory functions, is involved in the differentiation of myeloid and lymphoid cells [227, 248].

Although testosterone is often described to have an immunosuppressive effect, data on the effect of testosterone on vaccine‐induced immune responses are heterogeneous. Several studies found that testosterone is negatively correlated with antibody responses to influenza vaccination in males [223, 227]. However, in a study of healthy men receiving the inactivated influenza vaccine, Nowak and colleagues reported that males with higher testosterone levels produced more specific anti‐influenza antibodies following vaccination, conversely suggesting a potential supportive role of testosterone in adaptive immunity [249]. A positive correlation between testosterone and binding antibody levels was also observed in the case of recombinant DNA vaccination against hepatitis B (Engerix B) [250]. The interplay between sex hormones and antibody responses, therefore, remains inadequately understood and is specifically insufficiently investigated for next‐generation vaccines, such as mRNA or vector‐based platforms. Recently, Anticoli and colleagues [224] reported a positive correlation between receptor‐binding domain (RBD) specific antibody titers and testosterone levels in men after vaccination with the mRNA BNT162b2 vaccine. In contrast, another study reported negative correlations between testosterone and SARS‐CoV‐2‐specific antibody responses in males vaccinated with Comirnaty (BNT162b2) and/or Vaxzevria (ChAdOx1‐S) [251].

These inconclusive findings across influenza vaccines and other vaccine platforms underscore the complexity of the role of hormones and sexual dimorphism in vaccine‐induced immune responses. Further research is needed to further elucidate the role of sex hormones in shaping immune responses, particularly to next‐generation vaccines.

## 6 Sexual Dimorphism in Viral Vector Vaccine Responses: Challenges and Future Directions

Recent outbreaks of Filoviruses [252], SARS‐CoV‐2, and Mpox have highlighted the importance of next‐generation vaccines in combating emerging infectious diseases (reviewed in [5, 253]). As one of the vaccine platforms that can be easily adapted to combat emerging infectious diseases, viral vector vaccines provide favorable safety and immunogenicity profiles, inducing both robust humoral and cellular immune responses. Due to their excellent tolerability and safety profiles, the replication‐deficient viral vector vaccines are also safe for vulnerable populations, such as immunocompromised individuals [155]. Our understanding of sexual dimorphism in response to viral vector vaccines remains incomplete due to the disparities in the reporting of sex‐stratified data. This aspect was recently highlighted during the COVID‐19 pandemic, which provided a unique opportunity to comprehensively study sexual dimorphism in responses to various vaccine platforms targeting the same pathogen [30]. To date, sex‐specific immune responses after vaccination have been observed across preclinical studies, animal models, and in clinical trials [78, 180]. This indicates that sex bias in vaccine‐induced immunity can be detected early and may be important to consider already during the initial stages of vaccine development and early clinical trial design.

**FIGURE 2:**
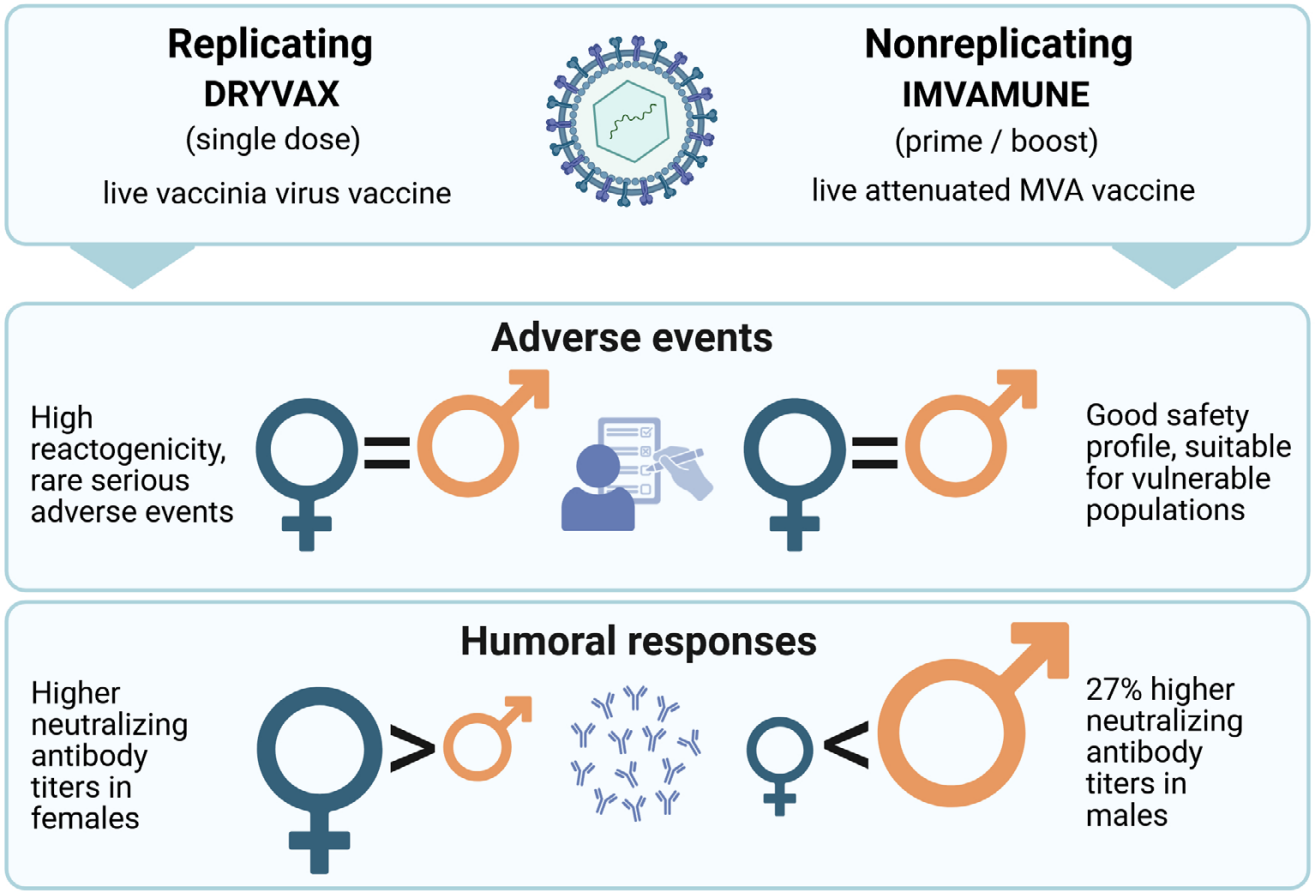
Viral vector vaccines against smallpox. Current knowledge on sexual dimorphism following vaccination with the replicating viral vector vaccine, Dryvax, and the nonreplicating viral vector vaccine Imvamune.

Emerging evidence suggests that biological sex leads to biases in vaccine‐induced immunity, with the magnitude and direction of these effects differing by vaccine platform and antigen delivery [15, 78, 80, 254]. While recent reports compare replicating and nonreplicating viral vector vaccines targeting the same pathogen, sex‐stratified data remain limited and mostly restricted to demographics [253]. An important insight into divergent sexual dimorphism following smallpox vaccination comes from studies of Imvamune and Dryvax [78, 80, 254]. These nonreplicating and replicating viral vector vaccines, respectively, suggest that the direction of sex‐related differences in immune responses may be associated with the vaccine platform and antigen delivery (Figure 2).

Current evidence suggests that the nonreplicating Imvamune has a more favorable safety profile than the replication‐competent Dryvax [255], and several reports indicate that sex is not associated with the incidence of adverse events [256, 257]. However, sex differences in humoral immunity in this specific context appear to vary by viral vector platform. While females demonstrate higher antibody titers after Dryvax vaccination [80], males exhibit higher antibody titers following Imvamune vaccination [78]. As reported by Byrne et al. [258], data on T‐cell responses remain limited, and to our knowledge, no studies have yet reported sex‐specific differences in cellular immunity after Imvamune administration. In contrast, a study on Dryvax revealed that male vaccinees demonstrate stronger IFN‐γ ELISPOT responses and increased IL‐1β secretion [254], opposite observations to the vaccine‐induced antibody responses described above. Furthermore, Haselow [256] found no association between adverse events or antibody responses with sex, age, or ethnicity following Dryvax vaccination. These inconsistencies may reflect biological heterogeneity within clinical cohorts as well as methodological differences in clinical trial design and selected endpoints. Taken together, evidence on Dryvax and Imvamune vaccines, two vaccines against the same pathogen, demonstrates the need for further research into sex‐specific differences in responses to replicating and nonreplicating viral vector vaccines.

Substantial gaps remain in our understanding of the mechanisms that drive sex‐based differences in vaccine responses and how these differences vary across vaccine platforms. Recent studies have demonstrated that vaccine‐induced immune responses may also exhibit assay‐specific and immune‐readout‐specific sex biases. As an example, following vaccination with a candidate HIV vaccine, women exhibited higher ADCC activity, whereas men exhibited higher HIV‐1‐specific CD4+ T‐cell response rates [2, 259]. Therefore, to gain a more comprehensive understanding of sex bias in response to vaccines, future studies should integrate multiple immunological assays, since sex‐specific patterns may vary depending on the immune readout investigated. Another challenge has recently emerged for nonreplicating viral vaccines, where the lack of balanced sex‐specific data continues to limit insights into sexual dimorphism. For example, in the case of mpox vaccination with MVA‐BN, most studies have focused on male populations, particularly men who have sex with men, based on the populations at highest risk in a specific disease outbreak context [258]. Consequently, it remains unclear whether these findings can be generalized to females. In addition, our overall understanding of sexual dimorphism in response to MVA‐based vaccines remains somewhat limited. An important effort to gain more evidence for this indication is the ongoing clinical trial in the Democratic Republic of the Congo (DRC) (NCT06844487), evaluating the immunogenicity of mpox vaccination (MVA‐BN) in pregnant women and children [260]. Adequate representation of both sexes and the inclusion of pregnant women in studies are crucial for deepening our knowledge of sex differences in vaccination and guiding public health strategies. Furthermore, growing evidence on sexual dimorphism in response to vaccines may offer future opportunities to improve vaccination strategies. For example, it has been shown that adult (18–49 years) females who received a half‐dose influenza vaccine developed comparable and sometimes even higher antibody titers to males who received a full‐dose vaccine [18]. This highlights the potential for tailored vaccine regimens that take sex and age into account, moving towards more personalized and cost‐effective approaches. In particular, reducing the vaccine dose for women could maintain protective immunity while reducing adverse effects, whereas increasing the dose for men could enhance antibody responses. These strategies demonstrate how sex‐specific dosing could optimize the efficacy and safety of vaccines and may offer solutions to address potential vaccine shortages, for example, in a pandemic context.

To conclude, viral vector vaccines may induce immune responses that differ from those of traditional vaccines (e.g., protein based vaccines), potentially resulting in distinct sex‐specific bias. To advance next‐generation vaccines and pave the way for more efficient, tailored vaccine approaches, future studies should systematically compare the available vaccine platforms and consistently report data in a sex‐stratified manner. Critical first steps are the consistent and standardized inclusion of biological sex as a key variable, and adapting clinical trial designs to better capture sex‐biased responses.

## Conflicts of Interest

The authors declare no conflicts of interest.

## Data Availability

The data that support the findings of this study are available on request from the corresponding author.
